# Percutaneous Surgical Treatment of Trigger Finger

**DOI:** 10.1055/s-0044-1788670

**Published:** 2024-09-04

**Authors:** Serdar Satılmış Orhan

**Affiliations:** 1 Clínica de Ortopedia e Traumatologia, Marmara University Pendik Training and Research Hospital, İstanbul, Türkiye

**Keywords:** tendons, trigger finger disorder/pathology, trigger finger disorder/surgery, trigger finger disorder/therapy

## Abstract

**Objective** To evaluate the efficacy of percutaneous release therapy for patients with trigger finger.

**Methods** We obtained the hospital records of 120 patients who underwent percutaneous release, and their final status was evaluated by telephone.

**Results** The sample was composed of 84 (70%) female and 36 (30%) male patients, with a mean age of 55.4 (range: 30–79) years, and a mean follow-up of 28.6 (range: 6–74) months. Successful results were obtained in 118 (98.3%) patients. In the first week after the procedure, release was performed through the open surgical method in two patients who had complaints of re-entanglement in their fingers. No limitation to the joint range of motion was detected in any finger.

**Conclusions** Percutaneous release has advantages over the open surgery method in the surgical treatment of trigger finger, due to its low cost, ease of application, performance outside operating room conditions, and similar complication rates.

## Introduction

Trigger finger is a common disorder that causes symptoms such as pain and snagging during finger movements. 1 Conservative treatment is recommended, especially in patients with mild-to-moderate symptoms. Conservative treatment includes the use of non-steroidal anti-inflammatory drugs, local corticosteroid injection, splint use, and physiotherapy and rehabilitation. 1

Surgical methods are preferred in cases that do not respond to conservative treatment or recur afterwards. Two techniques have been described: open and percutaneous surgery. It has been reported that the recurrence rates in release with the open surgical method are lower than with the percutaneous method. However, it is known that the percutaneous method is more advantageous in terms of length of hospital stay, cost, and scarring. 2 3 4

In the present article, we evaluated the efficacy of percutaneous release therapy for patients with trigger finger.

## Materials and Methods

### Study Design and Sample



**Video 1** Checking the location of the needle with finger movement in closed trigger finger treatment: We confirm that the needle is on the tendon because it moves with finger movement.


By scanning the hospital automation system, we obtained data from the hospital records of 120 patients who admitted to the Orthopedics and Traumatology Outpatient Clinic due to trigger finger between November 2016 and August 2022 who underwent percutaneous release. The final status of the patients was evaluated by telephone.

Patients over the age of 18, who had entanglement in the fingers and no infection, contracture, or other pathologies in the operated site, and who had been operated on by the same surgeon were included in the study. Patients under the age of 18 who had undergone previous surgery in the operated site were excluded from the study. The Green classification was used in the clinical evaluation of the patients. 5

**Fig. 1:**
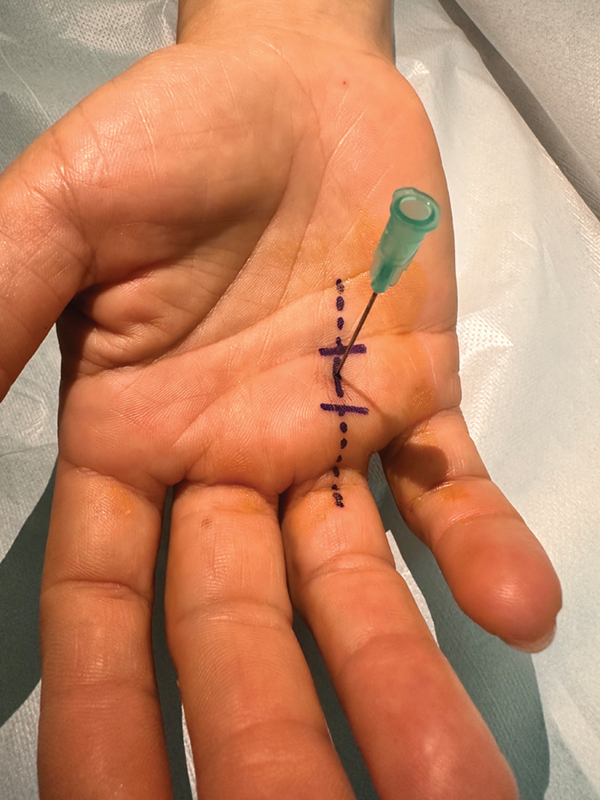
Topographic entry site of the needle on the A1 pulley.

As for the percutaneous surgical release procedure, the patients were informed about trigger finger and the treatment methods. Free and informed consent was obtained for the percutaneous release. The anatomical location of the A1 pulley was determined by considering the anatomical landmarks described by Wilhelmi et al. 6 and Sato et al. 7 In addition, the anatomical region was confirmed through the detection of the triggered area by asking the patients to perform active and passive flexion-extension movements of the finger under palpation. Subsequently, 2 mL of 2% prilocaine were injected under the skin over the A1 pulley. After anesthesia was provided, penetration of the A1 pulley and flexor tendon was ensured, with the sharp end of the 21-gauge (with 0.8 in diameter and 38 mm in length) needle parallel to the longitudinal direction of the tendon. Then, it was checked whether the needle was also moving by passively moving the patient's finger, and it was confirmed that the needle was on the tendon. Then, the needle was pulled back and removed from the tendon, the tendon sheath and A1 pulley were passed, and the A1 pulley was cut by moving it parallel to the tendon. While the A1 pulley was being released, it was felt that the needle tip penetrated the band/pulley and released it. At the end of the procedure, it was felt that the needle tip moved freely without friction against any band/fibrous structure. Then, the needle was withdrawn, and the patients were asked to actively flex and extend their fingers and whether there was any snagging. Afterwards, the surgeon made passive flexion-extension movements of the finger to check whether it was stuck or not. If there was no feeling of being stuck after the examination, the procedure was terminated by considering that the loosening was completed. The procedure was repeated in patients with ongoing complaints and persistent trapping of the tendon. After the release, the entry site of the needle was closed with a bandage for 1 day ( Video 1 and Fig. 1 ).

After percutaneous release, the patients were shown and asked to perform frequent movements during the day for 10 days, bringing the metacarpophalangeal, proximal and distal interphalangeal joints to full hyperextension and full flexion and holding them for a few seconds. The patients returned to the outpatient clinic for follow-up one week after the procedure. Information about the patients' range of motion of the finger joints, snagging, pain, and swelling was obtained by telephone interviews. To do so, we contacted the patients through the telephone number listed in their file. After verbal consent was obtained, we thoroughly questioned and recorded their current complaints. To evaluate the range of motion, we instructed patients to take a photograph of their fingers in both fully-flexed and fully-extended positions and send it to us. Patients who could not establish adequate and healthy communication over the phone (such as patients with speech and comprehension disorders) were required to attend the hospital for an outpatient clinic appointment.

This study was approved by the Clinical Research Ethics Committee at the Hamidiye Faculty of Health Sciences, University of Health Sciences, on October 16, 2022, under registration number 22/93. The study was conducted in accordance with the principles of the Declaration of Helsinki. The patients and/or their families were informed that data from the case would be submitted for publication and gave their consent.

## Results

The sample was composed of 84 (70%) female and 36 (30%) were male patients, with a mean age of 55.4 (range: 30–79) years and a mean follow-up of 28.6 (range: 6–74) months. The distribution of fingers and their laterality is presented in Table 1 . The dominant hand in 119 patients was the right, and only 1 patient's dominant hand was the left. According to the Green classification, 75 fingers were compatible with type 2, 60 fingers, with type 3, and 5, with type 4. Successful results were obtained in 118 (98.3%) patients. In the first week after the procedure, release was performed though open surgical method in 2 patients who had complaints of re-entanglement in their fingers. During open release, it was observed that the A1 pulley was cut incompletely, and there were small, superficial injuries along the longitudinal axis of the flexor tendon. Complications such as tendon rupture, infection, nerve injury and hematoma were not observed in any of the patients. It was observed that only 5 patients continued to complain of pain in the region of the A1 pulley. The complaints of these patients also regressed within 1 month. No limitation to the joint range of motion was detected in any finger.

**Table 1:** Distribution of fingers and laterality among the patients

Finger	Right	Left	Total	%
1.	31	29	60	42.86
2.	4	2	6	4.29
3.	16	13	29	20.71
4.	17	18	35	25.00
5.	8	2	10	7.14
Total	76	64	140	100.00
%	54.29	45.71	100.00	

## Discussion

Trigger finger is more common among women, especially in the fifth and sixth decades of life. The dominant extremity is more frequently involved. Furthermore, trigger finger is most common in the thumb, followed by the ring, middle, index, and little fingers. 1 Our data were also found to be compatible with those of the literature, including the age at disease incidence and gender distribution.

Green classified trigger finger into four types according to the flexion-extension movement. 5 According to the Green classification, conservative treatment methods are preferred in the early stages (types 1 and 2), and surgical methods are preferred in advanced stages (types 3 and 4). 4 8 9 The patients included in the present study were classified as types 2 to 4.

The superiority of the percutaneous surgical treatment over the conservative treatment or steroid injection has been proven in the treatment of trigger finger. 9 10 In the study by Zyluk and Jagielski, 10 in which percutaneous release and steroid injection were compared, the results of steroid injection were better in the 1-month short-term follow-up. However, recurrence was observed in 6 (11%) of the patients who received steroid injection in the 6-month follow-up, while no recurrence was observed among those submitted to percutaneous release, who obtained better results. In the current study, a success rate of 98.3% (118 patients) was obtained, which is in accordance with the data reported by Zyluk and Jagielski. 10

Although tendon and peripheral nerve injury can be observed with both surgical methods, inadequate release and damage to the flexor tendon are at the forefront in percutaneous surgery. 11 12 Wound issues and scar appearance are at the forefront in open surgery, but some authors 13 14 do not categorize these skin problems as complications. On the other hand, some studies have reported that tenderness on the scar may even lead to flexion limitation. 15 In the present study, open surgical release was only performed in 2 patients with inadequate release. The clinical significance of flexor tendon injury is not clear. We followed up our patients for a mean of 28 months, and no problems related to tendon damage were detected.

## Limitations

The weaknesses of the present study are that it is retrospective and does not have a comparison group. However, the long follow-up period, the sufficient number of patients, and the fact that it was performed by a single physician are the distinguishing features compared to similar studies. Prospective, well-designed studies with a comparison group are needed to reach clearer data on this subject. Another important limitation of the current study is that we did not use a visual pain scale. On the other hand, we chose to prioritize the presence of pain over its degree, as our investigation focused on the results of the percutaneous technique.

## Conclusion

In conclusion, percutaneous release has advantages over the open surgery method in the surgical treatment of trigger finger, due to its low cost, ease of application, performance outside operating room conditions, and similar complication rates.
