# Supplementary material for: Percutaneous Surgical Treatment of Trigger Finger
Source: Rev Bras Ortop (Sao Paulo). 2024 Sep 4;59(4):e580–3. doi: 10.1055/s-0044-1788670 (PMC11374397; doi:10.1055/s-0044-1788670)
Supplement: Related texts

# Research article: Tratamento cirúrgico percutâneo do dedo em gatilho

**Keywords:** dedo em gatilho/cirurgia, dedo em gatilho/patologia, dedo em gatilho/terapia, tendões

## Abstract

**Objetivo** Avaliar a eficácia da terapia de liberação percutânea em pacientes com dedo em gatilho.

**Métodos** Obtivemos os prontuários hospitalares de 120 pacientes submetidos à liberação percutânea, e seu estado final foi avaliado por telefone.

**Resultados** A amostra foi composta de 84 (70%) pacientes do sexo feminino e 36 (30%) do sexo masculino, com média de idade de 55,4 (variação: 30–79) anos e tempo médio de acompanhamento de 28,6 (variação: 6–74) meses. Bons resultados foram obtidos em 118 (98,3%) pacientes. Na primeira semana após o procedimento, a liberação foi realizada por método cirúrgico aberto em dois pacientes que apresentavam queixa de recidiva do quadro. Nenhuma limitação da amplitude de movimento articular foi detectada em nenhum dedo.

**Conclusão** A liberação percutânea apresenta vantagens em relação ao método cirúrgico aberto no tratamento cirúrgico do dedo em gatilho, como baixo custo, facilidade de execução, realização fora de centro cirúrgico, e taxas de complicações semelhantes.

## Introdução

O dedo em gatilho é um distúrbio comum que causa sintomas como dor e travamento durante os movimentos dos dedos. 1 O tratamento conservador é recomendado, especialmente em pacientes com sintomas brandos a moderados. O tratamento conservador inclui anti-inflamatórios não esteroidais, injeção local de corticosteroides, uso de talas, fisioterapia e reabilitação. 1

Os métodos cirúrgicos são preferidos nos casos que não respondem ao tratamento conservador ou que apresentam recidiva. Duas técnicas foram descritas: a cirurgia aberta e a cirurgia percutânea. Há relatos de que as taxas de recidiva da liberação após o método cirúrgico aberto são inferiores às do método percutâneo. Porém, sabe-se que o método percutâneo é mais vantajoso em termos de tempo de internação, custos e cicatrização. 2 3 4

Neste artigo, avaliamos a eficácia da terapia de liberação percutânea em pacientes com dedo em gatilho.

## Materiais e Métodos

## Delineamento Experimental e Amostra

A pesquisa do sistema de automação hospitalar levou à obtenção das informações dos prontuários hospitalares de 120 pacientes atendidos no Ambulatório de Ortopedia e Traumatologia por dedo em gatilho entre novembro de 2016 e agosto de 2022 e submetidos à liberação percutânea. O estado final dos pacientes foi avaliado por telefone.

Este estudo incluiu pacientes maiores de 18 anos com quadro de dedo em gatilho, que não apresentassem infecção, contratura ou outra doença na área operada, e cujo procedimento cirúrgico tivesse sido feito pelo mesmo profissional. Foram excluídos pacientes menores de 18 anos e os que já haviam sido submetidos a cirurgias anteriores na área do procedimento. A avaliação clínica dos pacientes ocorreu segundo a classificação de Green. 5

Quanto ao procedimento de liberação cirúrgica percutânea, os pacientes foram informados sobre o dedo em gatilho e os métodos de tratamento. Obteve-se consentimento livre e esclarecido para a liberação percutânea. A localização anatômica da polia A1 foi determinada considerando-se os marcos anatômicos descritos por Wilhelmi et al. 6 e Sato et al. 7 Além disso, a região anatômica foi confirmada mediante a detecção da área com gatilho pela realização de movimentos ativos e passivos de flexão-extensão do dedo durante a palpação. Posteriormente, 2 mL de prilocaína a 2% foram injetados sob a pele sobre a polia A1. Após a anestesia, a polia A1 e o tendão flexor foram penetrados com a ponta afiada da agulha de calibre 21 (diâmetro de 0,8 mm e comprimento de 38 mm) paralela à direção longitudinal do tendão. Em seguida, verificou-se se a agulha também se movia durante a movimentação passiva do dedo do paciente, e confirmou-se que a agulha estava no tendão. A agulha foi puxada para trás e retirada do tendão, a bainha do tendão e a polia A1 foram atravessadas, e a polia A1 foi seccionada ao ser movida paralelamente ao tendão. Enquanto a polia A1 estava sendo liberada, era possível perceber que a ponta da agulha penetrava a bainha/polia e a liberava. Ao final do procedimento, percebia-se que a ponta da agulha se movia livremente sem atrito contra qualquer faixa/estrutura fibrosa. Então, a agulha foi retirada e solicitou-se aos pacientes que flexionassem e estendessem ativamente os dedos e que indicassem se havia algum travamento. O cirurgião realizou movimentos passivos de flexão-extensão do dedo para verificar se havia ou não aprisionamento. Caso não houvesse sensação de travamento após o exame, o procedimento era encerrado, considerando-se a liberação concluída. O procedimento foi repetido em pacientes com queixas contínuas e aprisionamento persistente do tendão. Após a liberação, o sítio de entrada da agulha foi fechado com curativo por 1 dia ( Vídeo 1 e Fig. 1 ).

**Vídeo 1** Verificando a localização da agulha com o movimento do dedo no tratamento do dedo em gatilho fechado: confirmamos que a agulha está no tendão porque ela se move com o movimento do dedo.


**Fig. 1:** Local de entrada topográfica da agulha na polia A1.

Após a liberação percutânea, mostrou-se e solicitou-se aos pacientes que realizassem movimentos frequentes diários durante 10 dias, levando as articulações metacarpofalangianas e interfalangianas proximais e distais à hiperextensão e flexão completa e mantendo-as assim por alguns segundos. Os pacientes voltaram ao ambulatório para acompanhamento na primeira semana após o procedimento. Informações sobre a amplitude de movimento das articulações dos dedos, travamento, dor e inchaço foram obtidas por entrevista telefônica. Para tanto, entramos em contato com os pacientes por meio do telefone listado em seus prontuários. Após a obtenção do consentimento verbal, questionamos e registramos minuciosamente suas queixas atuais. Para avaliar a amplitude de movimento, instruímos os pacientes a tirar fotografias de seus dedos em flexão total e extensão total e enviá-las para nós. Pacientes que não conseguiam estabelecer comunicação adequada e saudável por telefone (como pacientes com distúrbios de fala e compreensão) eram obrigados a comparecer ao hospital para consulta ambulatorial.

Este estudo foi aprovado pelo Comitê de Ética em Pesquisa Clínica da Faculdade de Ciências da Saúde Hamidiye da Universidade de Ciências da Saúde, em 16 de outubro de 2022, sob o número de registro 22/93. O estudo foi conduzido de acordo com os princípios da Declaração de Helsinque. Os pacientes e/ou seus familiares foram informados de que os dados do caso seriam submetidos à publicação e deram seu consentimento para tal.

## Resultados

A amostra foi composta de 84 (70%) pacientes do sexo feminino e 36 (30%) do sexo masculino, com média de idade de 55,4 (variação: 30–79) anos e acompanhamento médio de 28,6 (variação: 6–74) meses. A Tabela 1 mostra a distribuição dos dedos e sua lateralidade. Em 119 pacientes, a mão dominante era a direita, e somente 1 paciente tinha a mão esquerda dominante. Segundo a classificação de Green, 75 dedos eram do tipo 2, 60 dedos, do tipo 3, e 5, do tipo 4. Resultados bons foram obtidos em 118 (98,3%) pacientes. Na primeira semana após o procedimento, a liberação pelo método cirúrgico aberto foi realizada em 2 pacientes que apresentavam queixa de recidiva do quadro. Durante a liberação aberta, observou-se a secção incompleta da polia A1 e a presença de pequenas lesões superficiais no eixo longitudinal do tendão flexor. Complicações como ruptura de tendão, infecção, lesão nervosa e hematoma, não foram observadas em nenhum dos pacientes. Observou-se que apenas 5 pacientes continuaram com queixa de dor na região da polia A1. As queixas desses pacientes regrediram em 1 mês. Não houve detecção de limitação da amplitude de movimento articular em nenhum dedo.

**Tabela 1:** Distribuição de dedos e lateralidade entre os pacientes

Dedo	Direito	Esquerdo	Total	%
1.	31	29	60	42,86
2.	4	2	6	4,29
3.	16	13	29	20,71
4.	17	18	35	25,00
5.	8	2	10	7,14
Total	76	64	140	100,00
%	54,29	45,71	100,00	

## Discussão

O dedo em gatilho é mais comum em mulheres, especialmente entre a quinta e a sexta décadas de vida. A mão dominante é acometida com maior frequência. Além disso, o dedo em gatilho é mais comum no polegar, seguido dos dedos anelar, médio, indicador e mínimo. 1 Nossos dados foram compatíveis com os da literatura, inclusive a idade à incidência da doença e a distribuição por sexo.

Green classificou o dedo em gatilho em quatro tipos de acordo com o movimento de flexão e extensão. 5 De acordo com a classificação de Green, os métodos de tratamento conservadores são preferidos nas fases iniciais (tipos 1 e 2), ao passo que os métodos cirúrgicos são preferidos em estágios avançados (tipos 3 e 4). 4 8 9 Os pacientes incluídos neste estudo apresentavam dedo em gatilho de tipos 2 a 4.

A superioridade do tratamento cirúrgico percutâneo com relação à terapia conservadora ou à injeção de corticosteroides foi comprovada no tratamento do dedo em gatilho. 9 10 Zyluk e Jagielski 10 compararam a liberação percutânea e a injeção de corticosteroides, e observaram resultados melhores com a injeção de corticosteroides no acompanhamento curto após 1 mês. No entanto, 6 (11%) dos pacientes que receberam injeção de corticosteroides apresentaram recidiva no acompanhamento aos 6 meses, ao passo que nenhuma recidiva foi observada nos indivíduos submetidos à liberação percutânea, que apresentaram resultados melhores. O presente estudo obteve uma taxa de sucesso de 98,3% (118 pacientes), o que está de acordo com os dados relatados por Zyluk e Jagielski. 10

Embora a lesão do tendão e do nervo periférico possa ocorrer com os dois métodos cirúrgicos, a liberação inadequada e o dano ao tendão flexor são mais comuns na cirurgia percutânea. 11 12 Os problemas da ferida e a aparência da cicatriz são mais comuns na cirurgia aberta, mas alguns autores 13 14 não categorizam essas questões cutâneas como complicações. Por outro lado, alguns estudos relataram que a sensibilidade da cicatriz pode até limitar a flexão. 15 No presente estudo, a liberação cirúrgica aberta somente foi realizada em 2 pacientes com liberação percutânea inadequada. A significância clínica da lesão do tendão flexor não está clara. Acompanhamos nossos pacientes por uma média de 28 meses, e não detectamos nenhum problema relacionado a danos nos tendões.

## Limitações

Os pontos fracos do estudo são sua natureza retrospectiva e a ausência de um grupo para comparação. Porém, o longo período de acompanhamento, o número suficiente de pacientes e o fato de o procedimento ter sido realizado apenas por um médico são os diferenciais deste estudo em relação a outros semelhantes. Estudos prospectivos e bem delineados, com grupos comparativos, são necessários para obter dados mais claros sobre esse assunto. Outra limitação importante deste estudo é a não utilização de uma escala visual de dor. Por outro lado, optamos por priorizar a presença de dor em detrimento do seu grau, pois nossa investigação se centrou nos resultados da técnica percutânea.

## Conclusão

Em conclusão, a liberação percutânea apresenta vantagens com relação ao método cirúrgico aberto no tratamento cirúrgico do dedo em gatilho devido ao seu baixo custo, facilidade de execução, realização fora do centro cirúrgico e taxas de complicações semelhantes.
